# Extracts from Correspondence

**Published:** 1846-07

**Authors:** 


### IV. EXTRACTS FROM CORRESPONDENCE.

[The following pages consist of extracts from a very voluminous and interesting correspondence with which the editor has been favoured since the publication of the article on homœopathy and allopathy, in the January number of this journal. They are made public, in the hope that they may tend to promote the object with which the article was written—viz. the improvement of practical medicine. Emanating, as they almost all do, from men not only of reputation, but of long experience—from men in every rank of the profession, and living in different countries, they will show that the opinions promulgated by the editor are neither novel nor singular, but are in strict accordance with those entertained by the most eminent members of our body. All the merit the writer of the article in question has ever sought to claim for it, is, that it openly avowed what the writer knew to be the sentiments of the wisest and best among his brethren. The correspondence and the other documents now published will, it is hoped, spread more widely the knowledge of the fact—that such are in reality the sentiments of a large portion of the physicians and surgeons of the present day. Such a knowledge cannot fail to give greater confidence to the younger members of the profession, not merely to declare their opinions, but to modify their practice according to the views they may conscientiously entertain. It is mainly to the younger members of the profession that the writer of the article in question looks for the consummation of the reformation in therapeutics, which he is desirous of promoting, and which he believes to be absolutely necessary —and inevitable.

In his desire to forward the cause in which he has embarked, the editor has entirely disregarded the imputation to which he has probably laid himself open in printing these extracts—that, namely, of publishing his own praises. In sending them to the press he has certainly omitted much that was complimentary: but if he had omitted all, he would obviously have defeated the object he had in view in publishing them,—the corroboration of his own views by the high authority of others. The editor will only further add, that as none of these communications were written with a view to publication, he has not only omitted the writers' names, but left out every indication of their precise locality. He trusts that his excellent friends and correspondents will forgive him for making the profession generally partakers in the great satisfaction and advantage which he himself has derived from their communications.]

---

*(A)* " ——— (America), January 30th, 1846.

"I thank you much for having written the article. The fulness of time has come in which all this matter should be exposed fully and clearly. You have not said a thing which I have not thought of and agreed to beforehand. I know how presumptuous this may seem; but you know that it is one thing to entertain correct thoughts on a subject, and quite another thing to bring them out clearly and in proper order, so as to claim the assent of all good judges. This last is what you have done most satisfactorily.

"Some of the opinions you have expressed I have entertained for many long years; others I have arrived at more lately. I first longed for a good natural history of diseases, to decide how far remedies had an effect. As connected with this I early learned the primary importance of diagnosis, and this in reference to the different stages of diseases as well as in reference to diseases themselves. It is many years since I was satisfied, in respect to acute diseases, that it was only on the first days (principally the first three days) that medicine

(drugs) could be of much service. At this period I am still satisfied that acute diseases can, ordinarily, be much mitigated and somewhat shortened. But an exception must be made in regard to such as we call malignant, (yellow fever, Asiatic cholera); and, as to shortening, in reference to the exanthemata. I have been getting more and more of the opinion, that in most chronic diseases, diet and regimen will often have a great influence,—drugs rarely any very decisive good effect, and often an injurious effect only. I have long deprecated the idea (and this I have done in communication with my intelligent patients) that medicines (drugs) are necessary in the treatment of all diseases. I have urged that it was the business of the physician to take care of the sick, pointing out that *cure* and *care* were the same word originally,—and that in taking care, it was much more important that he should endeavour to control the influence of the common agents, than that of the occasional ones, called medicines;—that he should attend to the non-naturals,—those things which nature does not decide, but leaves to our choice,—that he, the physician, should do it, and not leave it to the nurse or the grandmother. In consonance with these views, I have been unwilling to say that my patients who recovered were cured by me; for I endeavoured to cure all of them, and claimed to have done it, even when they died. In our hospital, opened twenty odd years ago, I would not allow the record books to say that so many patients had been cured, as is a common practice; but that so many were discharged well, so many improved in health, &c. I have often urged upon my brethren that we should never get the better of quackery so long as we attributed the recovery of our patients to medicine, on the *propter hoc* principle,—that is, *propter hoc* because *post hoc*. Our proper ground is, that, having studied the subject and had personal experience, we know better than others how to direct the cure of the sick; and that in doing so we may use drugs, or may not, as the case may require. These drugs may sometimes be directed against the principal disease; but oftener they may be used to counteract or guard against the accidents which would aggravate that disease.

"I have found no difficulty in all this. In some respects I have, perhaps, had advantages which you do not yet enjoy. . .Though we have good apothecaries, by whom alone medicines are ordinarily put up,—they are strictly men of the shop. They never visit the sick, and do not pretend to practice. They make their charges for medicines alone, and are paid as grocers or other tradespeople are. The physicians (and practitioners generally) charge first for their services whether they order medicine or not. Thus, there are none of the inducements to employ drugs which exist among a large class of practitioners with you. In our country towns the physicians supply the drugs; still the influence of city customs prevents their resting their emoluments on these; they charge distinctly and mainly for their visits, &c. Again, among the regular practitioners we have not any distinction of classes; though age, &c. give to some men a certain rank, it is very different from what exists with you. We all of us have therefore a chance to get patients at the onset of disease.........

"I have said that I have found no difficulty in satisfying patients without medicines. I began life with a good confidence in certain articles, and more especially in the alterative powers of mercurials. The doctrine now prevalent with you as to the power of calomel in acute diseases, especially in inflammation, was familiar to me, and fully believed, when I attended St. Thomas's, in 1799-1800. I used to say to my young friends there, with the true juvenile conviction of superiority, that it was singular Dr. Saunders (William) could be so blind; in his book, and in his lectures, he advised the use of mercury in acute as well as in chronic hepatitis, yet did not see that it ought to be equally useful in other acute inflammations, as I knew that it was. It was some years later that one of your Dr. Hamiltons (in England, not Scotland) first brought forward the general doctrine; at least, so far as I know. At the present day, I find it maintained by Dr. Watson in his delightful lectures. Now, I am not ready to say that the practice is all wrong. There is perhaps some good in it in some cases; but when pursued heroically it does a wonderful deal of mischief. Whether it often *causes* a fatal result I will not say; I think not often; but it often adds much to the suffering of the patient and to the duration of his *sickness* if not of his *disease*. I began to perceive this early in my career, but not in its whole extent for more than twenty-five years. In acute rheumatism I gave up the mercurials within six or eight years after I began; though it would seem to be the disease to which it should be most applicable. And as early as this I became very careful not to produce a bad sore mouth in all cases of disease. But I was a pioneer here; and I can boast (for I may seem to be boasting) much more of what has been done by younger men—whose modes of philosophising in medicine I have influenced in some measure—than of my own doings.........

"I must finish by saying, that I am often consulted by people from the country, as well as from the city, in chronic diseases, though I have ceased to be a busy practitioner; and that I find no difficulty in sending away a patient, after an hour's lecture upon his mode of life and conversation, without any prescription for medicine. In epilepsy and in phthisis, except so far as symptoms may require to be alleviated, I always tell the patient at once not to employ any drug to remove his disease. But in epilepsy, not evidently from organic disease, and in phthisis at an early period, I venture to say that much alleviation and benefit may be derived from care as to diet, exercise abroad, &c. Do not understand that there is a precise formula which I follow in these cases. I try to communicate principles rather than rules, and I find a large proportion of patients can understand me."........

---

*(B)* " ——— (America) March 26th, 1846.

"A few weeks since I had the pleasure to receive your remarks on Homœopathy, &c., with an accompanying note, requesting the opinion of those to whom it was communicated, whether harmonizing with, or discordant from it.

"The remarks I read with the deepest interest; they open to the eye a new and beautiful view of medical practice; beautiful, because it harmonizes with natural laws which we are well acquainted with, but greatly neglect.

"For forty years and more I have been called to attempt the relief of suffering by medical and surgical administration; the result of my observation has been that drugs are frequently more injurious than beneficial, particularly where the course of disease is established; the glimpses of truth which have occasionally enlightened me now shine out broadly in your philosophical treatise. I heartily congratulate you on your having the courage to be its author, and sincerely believe it will do more service in the improvement of medical practice than any publication of the present day."

---

*(C)* " ——— (America) Feb. 28th, 1846.

"The article has created quite a sensation here, and knowing well that it could be laid hold of by the homœopathists, and garbled as it has been, I was myself anxious that it should be reprinted in full, so that no permanent misrepresentation might exist. The favorable portion of your remarks has already been extracted by them (the homœopathists,) yet they have not concealed that you are no homœopathist, and have endeavoured to show that you are not consistent, by contrasting your admissions in regard to the reform produced by the practice of Hahnemann with your exposition of its absurdities. The whole article accords signally with my own views. In regard to the 'agenda, cogitanda, &c.,' I have scarcely an objection to make. Whilst I lived in ———, I was generally regarded as an 'inert' practitioner, because I did not practice the energetic and heroic treatment universal there; and since then my remedial agencies have been considered to belong to a 'masterly inactivity.' I apprehend that in the progress of life, every one becomes less and less active; is more and more disposed to attend to the 'divinity that stirs within us;' is less and less disposed to believe in the special adaptation of drugs to special morbid conditions; and more and more in the great principles of hygiene and therapeutics. With one single admission only would I hesitate to accord. You ascribe immense influence to Hahnemann as a reformer of regular practice. In this country, his doctrine and course of treatment have had but little effect on the 'regulars.' In the cities they have long become less active; but if any one is entitled to the credit more than another here, it is Broussais. Nowhere, not even in France, were his views so extensively embraced; and under their adoption, the excessive bleedings of the Rush School and the hypercatharsis in use everywhere were abandoned, and a more rational and milder system introduced. The good sense of observers of the day has also, I apprehend, had much to do in bringing about this salutary reformation."

---

*(D)* " ——— (Germany) April 15th, 1846.

"I beg that you will allow me to thank you for the article on Homœopathy and Allopathy, contained in the January No. of your Review. It must be hailed with the greatest satisfaction by all the members of our profession who have its welfare truly at heart, and have searched for the solution of the contradictions and riddles presented by all the orthodox works on the different branches of our science, either when studied in books only, or when compared with the statements of unorthodox schools or the results of practice. Your article must necessarily produce not only a great sensation, 'but it will lead to the most positive and the most beneficial results,' since it contains the enunciation of a principle which has before been hinted at by others, and which many, and myself among the rest, have had an 'Ahnung,' of, (to use a favorite German term,) but which none have dared to give clear and decided utterance to. The article in question made upon me the impression of an outburst of matured thought and long-suppressed conviction, which has broken forth with all the vehemence of an explosion, but it is not the explosion of gunpowder, but the powerful ejection of the first steam from the boiler, which at once proves the strength and workmanship of the engine, and gives promise of a long and useful career. The works of Dr. Combe, to which I with so many others owe much—I might almost say, the entire direction of my professional creed and practice in the path of hygiene—have prepared the way and rendered the adoption of your views a less difficult matter than it might have been, had they been published ten years earlier; and though I am convinced that you yourself would soften some of the expressions used in the above-named article, and remove stumbling-blocks which may offend tender consciences, the profession are indebted to you for the clearness and precision and courage with which you have pointed out what ought to be our aim, what must be the guiding principle in all our labours, if we are to be useful to humanity and satisfied with ourselves.......

"I have learnt to look upon the prevention of disease—upon hygiene in its most extended meaning—as the true aim of the medical man. I have seen enough both in hospital and private practice to feel disgusted—I may admit as much to you—at the authorized quackery even of intelligent and highly educated medical men. I have felt the opprobrium severely, which must be the lot of the profession so long as they shut their eyes to the true working of natural laws, and as long as they wilfully refuse to admit inferences, which though necessarily the permanent basis of the "curative" art, clash with received notions and traditionary prejudices. Truly has it been said that the real object of our science is less the healing of disease than the correct guidance of those that are healthy, and the interpretation of those laws by which healing κατ' εξοχην is rendered unnecessary. What more melancholy fact can be presented to the mere prescriber when he first enters upon the duties of his benevolent profession, with the enthusiasm of unsoured philanthropy, than the continual assurance of the Nestors of the profession that the greater our experience the more positive the conviction that we can *do* nothing? and it only proves the immense force of habit that, with such convictions, we do not more frequently see men quit a profession which, under such circumstances, requires a constant exercise of hypocrisy and a sacrifice of principle. But Sir, thanks to you, and to men like Combe, Chadwick, Clark, the young generation see the radiancy of a new light, that warms the heart while it illumines the intellect; and though their path still continues beset with dangers, they feel a firm footing, and the slough of despond is passed.

"One more remark on your essay, and I have done. The profession are deeply indebted to you, not only for your advocacy of the physiological school, but also for the truly conservative manner in which you have given vent to your ideas. I do not hesitate to say that there has been intellect enough among the members of the medical profession to have given birth to similar views at an earlier age; but the authors of new theories have invariably felt too much vanity, and consulted their passing fame too much, and have raised party feeling, and the baneful effects of party spirit. What is there in a name? We might almost say everything; and thus it has been that Galenism, Brownism, and all the other isms that have proved an ignis fatuus to many, have at least retarded the due development and progress of medical science, more from the factious opposition or factious adherence to the principles individualized by the name, than by any inherent faults of the systems.

"I beg to apologize again for intruding myself upon your notice, but it is to me almost an act of devotion to express my opinion and my gratitude to one who has conferred a most signal benefit upon the whole profession, and more especially upon its junior members. Whether or not I shall ever have an opportunity to avow my adhesion to 'the natural system' publicly, will depend upon a variety of circumstances. If you meet me in the field of literature, I trust that you may not have reason to refuse me recognition as one of your earliest disciples; at all events, I shall endeavour to follow out those principles which I feel convinced afford the only true standard and rule to guide our actions by. They are the moving power in my private practice, and I am guided essentially by them in my labours in the hospital."

---

*(E)* "——— (Germany) May 1st, 1846.

"One of the first points that the junior members of the profession will anxiously wish to receive advice upon in reference to the Natural History School, I conceive to be, 'how they are to observe,' in how far they are to continue to act according to the hereditary doctrines, and in how far they dare trust their own discretion in treading in the footsteps of Nature. It is no trifling matter for a beginner to be cast upon the sea of doubt without a sure beacon to steer his skiff by, and though you may assert, and perhaps rightly, that the public will not suffer, still some regard ought to be had for the mental distress of those whose energy is not sufficient to carry them through the dangers that beset all innovators, and still are possessed of sufficient 'ingenium' to feel that they are not in the right path. There is a great additional difficulty in England, from the unfortunate and disgraceful fusion of the apothecary and the medical practitioner; for let the 'pures' boast as much as they list of their influence and power, they do not, nor can they ever gain that general influence that is enjoyed by the surgeon-apothecary. The mere apothecary, (in the continental sense of the word,) cannot possibly interfere with the march of intellect in the medical profession, but so long as the medical man is expected by the bulk of the population to give his quid pro quo in a more tangible shape than that of a prescription or advice, he must prefer poisoning others to starving himself. The universal complaint of the apothecaries in Germany is, that much less medicine is sold now than formerly, and it was only the other day that a lady made the characteristic observation to me that, whereas, in former times, the apothecary was in the habit of sending her family presents of lozenges in elegant boxes at Christmas, he now scarcely had a bill to send."

---

*(F)* "——— (Germany) March 8, 1846.

[*Translation.*]

"Being compelled to write in haste, I avail myself of the German preferably to my bad English .... The good fortune I have had, ever since the commencement of my medical studies, of residing in large hospitals, necessarily familiarized me early with the natural history of disease, a thing somewhat different, it is true, from what we read in many, and not all uncelebrated works. I have arrived at the conviction that in inflammation and fever our drugs prove rather mischievous than useful; and that Nature has then to overcome both the disease and the evil effect of the said drugs. I need scarcely tell an experienced physician that, under this term 'drugs,' I do not comprehend simply-mucilaginous, gently-resolvent, mildly-aromatic, or very slightly-astringent decoctions or infusions. My objection is to the frequent employment of emetics, purgatives, drastic resolvents, mercurials; of cuprous, cinchonous, æthereo-resinous preparations. It has been, is still perhaps, imagined, that with such remedies, inflammation and fever are to be 'cut short,' 'advanced to a crisis,' 'to resolution,' &c. Calm observation at the bedside, an unbiassed review of circumstances, long practical study of pathological processes at the dissecting table, demonstrate the untenable nature of all those fancies which have, alas, been handed down from generation to generation. Let not Nature be thwarted; above all, let external influences be properly regulated, the instincts of the patient judiciously ministered to. Under this kind of treatment diseases are assuredly less complex in their course, and more fortunate in their termination; whilst the patients themselves are spared the distress inseparable from the use of substances for the most part so little germane to the organism. With all this, however, I do not intend to reject all drugs; in their application I am guided by a regard to physiological processes and conditions, and in the very few instances in which I know of specific remedies, I gladly avail myself of them; e. g. quinine, strychnia, belladonna, hyoscyamus, digitalis, — opium and morphia, — iodine, — mercury, — ipecacuanha? — tartarized antimony?? — &c. &c.???

"With respect to external remedies—in fever and inflammation, where we have to combat local evils, I allow none but physical indications to be my guide. In chronic disease, the dietetic treatment, in its most comprehensive sense, should unquestionably take the first place, although the empirical use of certain remedies must be retained, until a more intimate knowledge of the composition of the blood, of the nutritive process, of the metamorphosis of matter, of neuro-physiology, either warrants the older methods, or holds out new.

"The employment of the lancet I have almost entirely abandoned, ordering only, say, amongst 1500 patients, three or four venesections. The inference here may, perhaps, be simply this,—that in our time, for our nation, for my particular patients, phlebotomy had not been required; I can only say that I have cured all kinds of inflammation as well, nay better, without its aid; and I am entitled to make this declaration, and to appeal to experience, inasmuch as I see, annually, in my public practice alone, from 1400 to 2000 patients, besides a considerable number in my private practice; whilst during a period of fifteen years, I have had the opportunity of comparing the various methods of treatment practised in the different provinces of our Empire. Leeches I hardly ever employ, except—and that mostly to gratify the patient—for purposes of mere local depletion. Cupping I order sometimes, but upon the whole, very rarely. My rate of mortality will bear a comparison with that of my—perhaps more active—colleagues, without discredit; nor do my patients remain longer under treatment; in acute cases, in particular, I certainly do not lose more than the others. If I chose, I might perhaps be able to adduce proofs of the superiority of the simple treatment, but this I should deem inappropriate. Enough if it show no inferiority....

It is easy for an hospital physician to register splendid diagnoses, to describe severe cases, to record a series of violent symptoms; but for reasons easily intelligible, there is seldom in such cases a rigorous certainty as to the diagnosis. In like manner, the fixing of the cure or amelioration of the patient rests upon the personal judgment of the physician, and the books of the hospital written by himself; and he, doubtless, often loves his patient and himself sufficiently well to dismiss the case as cured, the moment the most striking and distressing symptoms are removed; but merely to receive the patient back again after a brief interval....

"What do I think of *Homœopathy?* Ever since I began to *practise*, I have regarded allopathy, homœopathy, &c., as historical facts. From both I have derived instruction; from the former perhaps most. I believe that a good physician, in earnest both with humanity and his profession, cannot swear allegiance to any one standard only. The pharmacology, and more especially the pharmaco-dynamics of homœopathy, have given the impulse for a thorough reconsidering and weeding of the Materia Medica. Without entering upon the absurd mystifications of the ultras, both deceivers and deceived, I affirm that we have not a little to thank homœopathy for, more especially in relation to the so-called specifics, and also as to the lessening of our doses....

"The foregoing will show you the sort of value which *I* attach to the reports of hospital physicians, who contend exclusively for this, or any other system; *unconditionally, they are absolutely valueless.*

"The world—the crowd—the ignorant, (and how many physicians, some of high renown, might count among the number?) never can and never will judge correctly; nor will they ever be taught by us. We must leave the finishing of the battle—the triumph, and the union, if achievable—to time. I, for my own part, have resolved to let my creed be corroborated, more by deed than word; by actual experience, and not by written dogma."

---

*(G)* "——— April 29th, 1846.

"Lord Bacon says that 'It is not St. Augustine's, nor St. Ambrose's works, that will make so wise a divine as ecclesiastical history thoroughly read and observed;' and it was wisely said—for there he will have a history of the effects of ignorance and weakness, as well as of the art and ambition of human nature; and if a review of them does not make him a more charitable and wiser man, he must have a weak and highly prejudiced mind, or has it contracted and blinded by interested motives. An equally important lesson may be learned from the history of medicine; for there the physician will see the folly, credulity, and impositions which have been connected with the healing art; how the opinions and practice of its professors have fluctuated; and the conflicting testimonies of men all equally honest and zealous to promote the advance of medicine, and all equally positive as regards the effects of opposite modes of treatment in diseases. One would naturally conclude that the review of such a history would excite in the mind of a reflecting man a spirit of caution and modest doubt, in all cases where he had not the guide of uncontroverted facts and sound experience to justify confidence, decision, and assurance. There is nothing in the history of medicine, either in ancient or modern times, calculated to excite more surprise than the absurd statements connected with homœopathy and the astonishing credulity of the advocates of it. But if, as you conclude, and I believe justly, there are really honest men who sincerely believe in the whole of homœopathy, what a lesson should it teach us of humility and charity, that honest minds in the search of truth should embrace such follies!

"The practical importance of the statements made by the homœopathists is, that they furnish some aid in solving the interesting questions—how far the use of drugs and other active agents have tended to promote the cure of diseases? and how far, and in what respects, the present practice of medicine is preferable to that of Hippocrates, who chiefly relied on regimen and what is called the expectant treatment? Every medical practitioner, who is a friend to truth, and who wishes that medicine should be based upon rational principles, will court for its practice the strictest investigation and scrutiny, and rejoice to see it cleared of everything which is injurious or useless, that ignorance, prejudice, or credulity have introduced into it. It will be only persons to whom the words of Bacon are applicable who will fear the light of truth: 'Doth any man doubt that if there were taken out of men's minds vain opinions, flattering hopes, false valuations, imaginations as one would, and the like, but it would leave the minds of a number of men poor shrunken things, and unpleasing to themselves.'

"Nothing can be more injurious to the true interest of medicine than holding out false hopes in attributing too much to its powers. The power we possess in what are termed surgical cases is very different from that which we have over internal diseases; the changes which take place in our internal organs in disease are often of a mysterious nature, and out of our power to discover or control; and it is happily ordered that, in most cases, much more depends on the powers of Nature than the knowledge and skill of man, or else, woe unto the poor and helpless, and those who depend upon the judgment of the unskilful practitioner. But when the Healing Art has been stripped of all its unjust pretensions, much will be left to prove its importance. No one will deny the great improvements which, in modern times, have taken place in the treatment of cases which belong to surgery, and the immense benefit which surgery has conferred on mankind. It is equally certain that there have been great additions to our knowledge in regard to the remote and exciting causes, as well as the nature and treatment of internal diseases. If medical treatment only saved one life in a hundred, medicine would be a science of vital importance; and the man who does this surely should be as much valued as the pilot who, in conducting ships through a dangerous passage, preserved one in a hundred from destruction.

"There is no doubt that many practitioners of medicine have presumed too much on its efficacy, and, by an officious interference with Nature, instead of affording relief and benefit, have often been the cause of torment and injury, and thereby lowered the just estimation of the profession of medicine. There is as much truth as wit in Molière's description of the orthodox physician in his time: 'C'est un homme tout médecin depuis la tête jusqu'aux pieds; un homme qui croit à ses règles plus qu'à les démonstrations des mathématiques, et qui croirait du crime à les vouloir examiner; qui ne voit rien d'obscur dans la médecine, rien de douteux, rien de difficile; et qui, avec une impètuosité de prévention, une roideur de confiance, une brutalité de sens commun et de raison, donne au travers des purgations et des saignées, et ne balance aucune chose.' It will be allowed that the character of physicians has improved since the days of Molière, but if a little more 'modest doubt,' which has been called the beacon of the wise, had influenced their opinions and practice, the stability and dignity of medicine had not been left so much at the mercy of homœopathists and other theorists.

"One great cause of the present imperfect state of medicine is the ignorance of the public as regards the important principles which ought to guide the medical practitioner: they are pleased with what appeals to their senses rather than to their reason. The *opus operosum* of medicine has, in every age, been more captivating to all classes, than the recommendation of simple means which reason and experience may dictate as best adapted to their case. When Elisha told Naaman the leper to wash in the Jordan for the cure of his disease, he was wroth at the simplicity of the remedy, and went away and said, 'Behold! I thought he would surely come out to me and stand and call upon the name of the Lord his God, and strike his hand over the place and recover the leper;' and such unfortunately is the state at present of the public mind, that, in the majority of cases, a medical man cannot do justice to himself or give satisfaction to his patients, if he does not practise a little of the 'Art and Mystery' of Medicine.....

"Much has been said and written about medical reform, but in my opinion none is so desirable or would contribute so much to the welfare of the medical profession, as an elevation of the mental character of its members: this, I think, would be the best, if not the only remedy for the present defects and follies connected with the practice of medicine. There should be required from all those who enter the medical profession, a knowledge of authors whose works tend to develop the reasoning powers—such as those of Bacon, Locke, Paley, &c., as well as a knowledge of the history of medicine, and of subjects immediately connected with the preservation of health—public and private hygiene, in addition to those on which candidates are now accustomed to be examined. The first examination, (that qualifying for practice), might be of such a kind as any one of average talents, with common industry, may undergo with facility. Every one might follow that branch of practice which his talents, his taste, or circumstances may dispose him to cultivate; but surely no one will deny that a knowledge of surgical subjects is highly desirable, if not necessary, to the 'pure physician.' Professor Alison, of Edinburgh, says, that many physicians of high eminence, both those employed in consulting practice as well as those attached to hospitals, have few opportunities of witnessing the effects of bleeding in the early stages of violent inflammatory diseases (Pract. of Med. p. 221), and this observation is equally applicable to other important diseases. When a medical practitioner has had opportunities to acquire experience, and seen what may be called *the active service of medicine,* he would in the course of years, say ten, have some claim to the title of doctor, which he should acquire by undergoing a more extensive examination on all those subjects he had been previously required to have some knowledge of as a licentiate; and he might take his degree either as doctor of medicine, or of surgery, or both. If a competent knowledge of philosophical subjects, including mental and moral philosophy, was required of every one who took rank higher than a licentiate in medicine and surgery, it would give a motive for and a stimulus to the study of subjects the knowledge of which necessarily tends to elevate the character and accomplish what Hippocrates says ought to be done, 'to bring medicine to wisdom and wisdom to medicine, so that the physician should be a divine philosopher.' (De Decent. Habit.) Such a reform in my opinion would remove what are now thought to be unjust privileges and invidious distinctions, and tend to place the practice of medicine on the basis of sound experience and philosophy."

---

*(H)* "——— Hospital, 3d March, 1846.

"The general error into which English practitioners are falling is the empirical use of powerful remedies, with a strong disinclination to be quiet, even when the diagnosis is obscure. The general scope of your paper I take to be to combat this mistake, and I hope that it will be the means of bringing about an improvement in our practice. The table (Fleischmann's) you have given is curious, and could it be implicitly relied on, would prove to me at least that we had better, in some diseases, give up prescribing altogether. Thus we have 188 cases of rheumatism, all of which are cured; and even of articular inflammations, 203 are cured out of 211. Now we have no success at all approaching to this at our hospital. I treat about 100 cases of these diseases annually; but I should be ashamed to place the results in a tabular form by the side of the homœopathist's table.

"In regard to pneumonia, used as a general term, the results of our hospital practice would differ little from the table, or even appear more favorable. I studied under a physician of the old school, who was fully impressed with the virtues of Seneka in cases of inflammation of the respiratory organs, and seldom prescribed venesection; yet he was as successful as his neighbours. But I have so often witnessed the sudden extinction, as it were, of incipient pleuritis, or pneumonitis, by a timely bleeding, that I should consider myself culpable in neglecting such a remedy. The fatal cases of pneumonitis are those in which the disease has been silently going on for a week or more, without pain or much uneasiness, and the patient has been living in his accustomed manner, and perhaps taking more strong drink than usual to relieve the languor which he feels. We receive five or six such cases annually, mostly men who are living with their families, and are not seen daily by a medical officer. They are sent to us either as cases of typhus, or of pneumonia, with typhoid fever; but, in fact, they are cases of hepatized lung, proceeding to the gray or suppurating stage, and the great debility, which generally comes on suddenly, is caused by the want of oxygenization of the blood. They do not bear bleeding at the arm at all; and the blood drawn has a dark venous colour. This form of the disease is, I believe, more frequent with spirit drinkers.

"Again, of dysentery, all are cured in the homœopathic table, except two that died. Now, I have very many cases of dysentery from the coasts of Syria and China, and experience leads me to believe that permanent recovery is an exception to the general rule in cases sufficiently severe to require hospital treatment, after two or three months' duration. The cases discharged from the hospital are merely relieved, for the complaint recurs again and again, or alternates with irregular remittent; yet, judging from the result of dissections, there is no ulceration in the intestinal canal in the majority of fatal cases.

"Pericarditis, too, is set down as cured, in the only two cases mentioned in the table. It is a frequent disease with us, as might be expected, since rheumatism is our most common acute disease. Now, unless it can be speedily cut short, we find that the functions of the heart are permanently injured, and life shortened. A considerable number of men are invalided annually for this cause, and many of them return to the hospital in a year or two with the usual dropsical symptoms that attend dilatation of the ventricles of the heart in its advanced stage. In regard to continued fever, perhaps the homœopathic treatment is equal to any. When the skin is kept cool, thirst allayed, the bowels cleared out, and air freely admitted, the *vis medicatrix* must do the rest.......

"I trust that your paper will have a beneficial effect, by causing medical men to weigh the facts well before they come to a decision as to the effects of the medicines prescribed. I firmly believe that the cure is much oftener retarded by the medicines administered than it would be safe to say in these times of advanced medical knowledge, and the mischief would be still greater were it not that much of the stuff sent to the patient is not swallowed by him. Could the public mind be so far influenced that a post-mortem inspection of the body took place in every case of death, by experienced anatomists, the foundation of rational medicine would be laid. The opportunities we have of testing our diagnosis in hospitals, though not so good as they might be made under better arrangements, are of much service to the individual practitioners in charge, and might be made of more general use by a system of reporting."

---

*(I)* "——— February 18th, 1846.

"During above thirty years of extensive practice, consequent upon eight or nine years of studentship, I have really attended as unremittingly as most men to my profession; and am arrived at the period when I feel no hesitation in stating, that the best part of medical science consists in observing symptoms, so as to form a correct diagnosis, and in ascertaining the *restorative powers* of the system, so as to aid without interrupting *them*. I have long taken leave of the worrying excessive method of treatment, by which the juvenile practitioner expects to knock down every disease, as systematically and surely as the soldier the enemy's stronghold in a bombardment. I suppose most men, as they go on in years and experience, leave off the over-active dosings and depletions in all ordinary medical cases, and find themselves proportionately rewarded with success. I feel convinced that, in my own circle, I have witnessed patients evading successfully from a protracted and dangerous disease, owing to forbearance in the treatment of its early stages, who would, under an opposite plan, have shown no powers of recovery, or have sunk under diseases induced by the treatment.

"In proportion as a physician respects the natural *vis medicatrix*, does he attend to diet, exercise, fresh air, cheerful impressions, &c. which are admitted to be beneficial under all the theories and vagaries of practice. I have been so impressed with the effect of the mind upon a great proportion of the ordinary ailments claiming our attention, as to be led to remark, that the practitioner of great renown often gives a fillip to recovery by the confidence he inspires, and which the familiar and daily medical attendant has no chance of inspiring. You will readily conceive, my dear sir, that, embued with these sentiments, I can readily appreciate your explanation of the only merit and efficacy of homœopathy—the most nonsensical and wild doctrine that ever took possession of the human mind, not excepting the darkest period of our history—and when the practice is successful, this can only be attributed to the natural *vis medicatrix* having fair play in the absence of treatment by medicines, or to the favorable influence of the mind under the confidence inspired in the patient, or to both these, aided however by rules of diet, exercise, &c., which the homœopathists, I believe, pay sufficient attention to.

"I trust that your prediction, as to the homœopathic practice causing the restorative powers of the living system to be better understood and more respected, will be realized, and shall be glad to find that your pen continues to be exercised towards the fulfilment of so desirable an object. I am sure we want something to help us, and to right us, in this respect—for excessive bleedings, continual raking of the bowels by purges, and indiscriminate mercurialization of the system, cause more diseases than they cure. Injurious as these measures prove, unless temperately and very carefully applied, they are nevertheless regarded by those fresh from the schools as the great and chief resources against the most frequent diseases. I involve my own past errors and early notions in making this statement; and am free to confess that what I now know, in regard to the proper and successful treatment of many chronic diseases, and particularly such as require a tonic plan of diet or medicine, I have found out by experience, and was ignorant of them at first starting in practice."

---

*(K)* "——— (Scotland) 18th Jan., 1846.

"I have not ventured to bore you with my opinion of your 'Young Physic' article, because abundance of more valuable opinions would be poured in upon you for some weeks. Now, when probably the inundation of letters is diminished, or diminishing, allow me to say that I think it a right bold, and right good essay. On this side of the Tweed we all belong much more to the school of 'Young Physic' than our Southern neighbours, with all their gigantic drug-loving, drug-giving, and drug-swallowing propensities. Still, however, the article will be of great use even in old Scotland. Some here are in great wrath at it. Never mind. *Fiat justitia*, &c.

"Pardon me if I say I think you would have made the article as useful as it will be, and less offensive to the 'some,' if you had done two things—first, not *contrasted* Homœopathy and Allopathy, but merely adduced homœopathic results to show (as they do) medicines of no use in many recoveries; and, second, I think you should have brought out more forcibly the fact that we have some POSITIVE agents in allopathy—that opium does and can act as an anodyne, antimony can sweat, ipecacuanha vomit, jalap purge, &c. &c. What we want is more precision as to when and where we should purge, vomit, sweat, &c., or where we should *not*."

---

*(L)* "——— Feb. 7, 1846.

". . . . I do not imagine that my opinion will be of any consequence to a veteran writer like you, but I hope I may be excused for indulging myself in the pleasure I feel in expressing to you the great satisfaction with which I have read the article in question. It is long since I read anything embracing so many opinions on different subjects, to which I could give so hearty and unreserved an assent. It is a bold, a well-timed, and a classical exposition of most momentous truths; and, if it be appreciated, it may lead to most important results. I wish the spirit which dictated that article could be deeply impressed on the mind of every medical man in existence. Many of the opinions you have expressed I have often inculcated in my daily public duties; and I have so earnestly longed to see them more widely disseminated that, if my pen had been as ready as yours appears to be, or my engagements less pressing, I might have robbed you of the honour you have nobly achieved in thus giving them to the world.

"I expect the article will be extensively misunderstood, and your first return for it may not consist of unmingled praise; but you have raised a standard about which all right minds must sooner or later rally.

"Apart from lessening the mortality of diseases, we allopathists *may* do much good by shortening their *duration.* What you say about the cure of pneumonia (one of the most fatal internal inflammations), by the natural powers, I have often verified. I can also assent to what you say about the want of evidence of the powers of mercury in this class of inflammations."

---

*(M)* "——— (Scotland) 18th Jan., 1846.

"Your article in your last Number has stirred us like a trumpet. Many are terrified and enraged, as well as awakened, and, like 'Demetrius and the *craftsmen*' of old, are 'crying some one thing and some another;' for they are 'confused' as well as confounded. Our friends (the minims) are quite jocose and lively at being handsomely killed, decently buried, and having a sort of funeral service read over them. They are preparing, however, Phœnix-like, to rise from their own remains. You have done a great service to the profession and to mankind by giving them (in their present form) their quietus. But as to create is better than to destroy, you must go on to the *instauratio magna,* and give us, or help us to the giving of, a new *Organon,* in which Bacon's (because Nature's) rule will be followed, and the *end* of the healing art will be stated to be, not the knowledge of diseases, but the cure of them, by *knowledge* (of a certain kind, of course); but the knowledge as a means; the cure as the *end,* and the only end, though not the only result.

"Some of our better men, and who have been *lying awake, waiting to be called,* think your notice rash, and rough, and exaggerated. I say to them, a trumpet sounds an alarm, gives a challenge, calls attention, and signifies a position. It must be loud, it must be startling; it can hardly fail to be a little too loud, or a little too rough-voiced. I for one acknowledge it as a true and certain sound, and am ready to join the ranks; and my object in using the liberty I now do with you is first, to thank you most sincerely as a man and as a healer, for what you have done to *me* in this matter; and, secondly, to offer my services to you as a fellow-soldier and a free man, and one who serves the same queen (the Vis. Med. Naturæ).........

"We are all against the young giant being called 'Young Physic.' He must not be *nicknamed,* and by his own father too! We have got the *thing*—the *name* will come in due time.

---

*(N)* "May 21st, 1846.

"With regard to the soundness and the importance of the step you have taken, and been the first to take publicly in this our 'reformation,' I have even a stronger opinion than when I first wrote you, and feel personally grateful to you for the good I have already got from its effects upon my mind.

"I am delighted with your second explanation, and with the wise and pregnant letters of Dr. Combe; but I have a special affection for the original blast. Its true value will be better known, and more fully acknowledged, when you and I have been a hundred years in our graves.........

"I wish some one would do justice to Sydenham, and show how truly he was a 'minister and interpreter' of the Vis Medicatrix. I have long thought that something valuable, and at the same time entertaining, might be made of the three greatest practical healers we know, Hippocrates, Sydenham, and

John Hunter—men who combined the speculative with the practical, the deductive with the inductive, in rare quality and measure. Their lives and their doctrines and practice might be made to illustrate three great epochs of our art, and there might be a prognosis attempted of what is likely to be the fourth epoch, and what sort of man the fourth, should and is likely to be. But this subject, to be well handled, must be a work of time, and much labour and judgment."........

---

*(O)* "——— Jan. 11, 1846.

".... The conclusions you have arrived at meet with my cordial concurrence. For several years past I have been very much of an expectant practitioner, and I have no reason to be dissatisfied with the result, so far as my patients are concerned; but in private practice it is no easy matter to follow always your own judgment—so wedded are the *laity*, generally, to an opposite and *telling* system. I was educated by Dr. ——— in the strong-remedy method; but long previously to his death he was aware of the total change that my experience had effected upon me, and he did not express himself as if he believed I had gone wrong. When my views were a little strengthened by a still longer experience, I relinquished the plan of keeping medicines and preparing my own prescriptions; and I cannot but recommend my friends to do the same—so much comfort is there in this, and it removes the charge of tradesmen from us, and undoubtedly has a tendency to add to our respectability in the eyes of the public."

---

*(P)* "——— (Ireland) 13th Jan., 1846.

"I have carefully read your 'Young Physic' with much interest, and have taken every opportunity of eliciting the opinions of my brethren respecting it. Many quite coincide with your views; many deprecate not—so far as I could make out—your *views*, but your *article;* their disapprobation summing itself up in the complaint of the Ephesian of old, 'Sirs, our craft is in danger,' and bitterly complaining of the mischief and danger that might accrue should the *public* become indoctrinated with such principles. For my own part, I think all your positions are in the main correct; I only doubt that you have extended some of them too far. I verily believe, and have long believed, and have long taught, that the greater portion of our so-called curative methods are, to say the least, of doubtful efficacy; and, in my mind, the instances in which giving medicine and recovery from disease are satisfactorily connected as cause and effect are few compared with the mass of cases of disease, or rather of diseases, treated. But still, I think that in many and important instances such connexion does exist, and is known. The only question is to determine to what extent this is the case, and how such determination is to be effected. It may be that the only way is to make a *tabula rasa*, and begin the study of disease afresh—first, by ascertaining from observation the natural history of disease, and then determining, by experiment, how far we can modify disease by interference. This may be the case; but I am strongly inclined to believe that by consulting the records of experience we shall find the practical question decided, or at least we shall find the elements for deciding it, in some cases at least, or at all events, get a starting point, whence we may go on without commencing absolutely *de novo*. Thus, for example, take any acute disease which has been treated according to three, or four, or more methods, each of which has yielded the same, or *quam proxime* the same results. Let us, then, examine what these several methods of treatment possess in common with each other, and, excluding everything but what is common to them, treat the disease in a certain number of cases by this common term. If the result is the same, the problem is, I think, solved for this particular case. If it is better, the problem is solved in a still more satisfactory way. If it is worse than the different modes, its treatment must contain in the parts of each omitted, some one common perturbing influence—common, that is, in its effects; and it would remain to inquire what that perturbing influence was, and to apply it in its simplest form. As to chronic diseases, I almost despair of anything very definite being ever known respecting the true value of curative proceedings in most of them, because of the great length of time over which the experiment would extend in very many of them.........

"I think you must '*go on.*' If you, or some one else, does not *go on* in this direction, medicine is, I think, in danger of being utterly prostrated as a science and as a profession, and must inevitably descend lower and lower. It is now much, and promises to become still more, in the hands of the drugging and drenching branch of the profession; but the next slide downwards has already not only commenced, but has made considerable progress—the slide, namely, of the practice of medicine into the hands of professed nostrum vendors, on the one hand, and mesmerists, hydropathists, homœopathists, &c., on the other. If the regulars do not in time adopt the 'common term,' so very clearly indicated by the results of the practice of the latter-mentioned race, and adopt, in the matter of 'drugs,' *ne quid nimis* for their motto, *c'est fait de nous.*"

---

(*Q*) "Jan. 16, 1846.

".... I think you have expressed accurately the sentiments which prevail amongst the older practitioners, at least, you have expressed mine. It appears to me that under the new order of things which must evidently become established, the homœopathist will have a considerable advantage over the allopathist; his positive faith in his own remedies, if he has it, will enable him to sustain the confidence of his patient throughout a long and trying illness, whilst the negative faith of the allopathist will make it difficult to inspire his patient with such confidence as shall render him willing to trust to nature, and able to resist his own prejudices and those of his friends in favour of more active treatment. How is this to be remedied?"

---

*(R)* "——— Hospital, Feb. 15, 1846.

"I have read your 'Young Physic' and detected your hand at once. I have gathered the opinions of most of the profession here upon it. Their amount of approval has been in about an exact ratio with the degree of their knowledge and ability, as far as I am able to judge of the latter. There are some who think that you undervalue the powers of medical treatment, and that they have saved numbers of lives by various plans which others consider injurious. But all the best informed men here are delighted with your exposition of doctrines to which their own reflections had for years been leading them. As to myself I regard the article as just what I should have wished to write. I was for the first three years of professional life with Mr. ———, who had an immense number of patients, and, with my principal, placed most implicit confidence in the powers and virtues of the medicines we ordered. This was very pleasant if the patients got well, but I can answer for most painful feelings and anxiety if they did not; often attributing to our want of knowledge in treatment what I now know to be inevitable doom. I then was assistant for a year and a half with a thoroughly educated and clever man, Mr. ———, of ———, who first opened my eyes to the natural course of disease and the trivial effects of remedies generally used. Then I went to Dublin, and returned a little to former views in polypharmacy. Then I came to London, and under the late Dr. ———, of St. Thomas's Hospital, began to watch disease in, I hope, a more philosophical spirit, and have ever since been advancing towards the doctrines of 'Young Physic.' I need not say more than that I am sure your paper will be appreciated by the better class of the profession, and by most thinking men out of it."

---

*(S)* " ——— February 5, 1846.

"I have read your article on homœopathy with unmixed pleasure. It has created no small astonishment among the polypharmacists of the provinces, but all well-educated physicians will I am sure agree with you. You hit the homœopaths hard, and the more so because your eyes are so evidently open to the miserable drugging system, which ruins our practice on this side of the border. Of all countries, England is that which requires such a reaction as homœopathy, to bring the drug-doctors to their senses, and I rejoice to find you fully alive to the negative benefits thus bestowed by homœopathy, while you utterly destroy by your arguments that most absurd and baseless theory."

---

*(T)* " ——— January 23d, 1846.

"With regard now to your proposed reformation of physic practical, in contradistinction to physic political, I hardly know how to begin. The subject is so extensive, and my own ideas so crude and undigested, that I shall now attempt to say little more than that I feel there is abundant room for reform in allopathy, and that you have certainly strongly and strikingly pointed out its most glaring defects, and by thus affording food for reflection and consideration, have given a clue for the elaboration of some clear and definite ameliorations. In doing this I think you have been too sweeping in your general condemnation of practitioners. I cannot think that the extreme practice which you so strongly and justly condemn, is in this day adopted by well-informed and sensible men, and it is hardly fair to include them in the same category with the illiterate and unthinking mob. Still I am not sorry to find you dealing an unsparing measure, because nothing rouses a thoughtful man to self-examination and reflection more than a home charge, even though in great measure unfounded as regards him individually. After all, to the question *cui bono?* What should we be at? I am at a great loss to say in what direction the reform is to begin, for this simple reason, that though we have no right grounds for rejecting *in toto* the accumulated observations of past times, still the very foundation of our fabric requires investigation and thorough repair; and where are the minds to be found to do this? Where is the simple love of truth to be found which shall rightly and justly discriminate between fact and fiction—between truth and error—between prejudice and observation? Find your man or men who shall be both capable and willing to undertake such an investigation, when so many and powerful temptations are against them. Then comes the almost more difficult question—where is to be the field of their observation? Private practice never could afford it; and where is the hospital which would be sufficient as the theatre of experiments, the utility and real benefit of which, to the patients themselves and to the public, would be too remote and uncertain to be appreciated by the latter, or allowed by the former? I despair entirely of any general plan of co-operation, where the labourers are so generally, almost universally, unfitted for such a species of investigation. Those who can do much are fettered. Those who are willing for the race have no legs for it.........

---

*(U)* "——— Feb. 2, 1846.

"On the subject of the present state of therapeutical knowledge and practice, the great point seems to me to inculcate the necessity of giving up empirical prescribing, and as far as possible prescribing and treating upon the general broad principles which have been laid down and ascertained as truth. We want men who can take a comprehensive view of diseases and their management, who will make diagnosis a more prominent feature in the training of their minds for practice, and who, when for want of an accurate chart they are obliged to sail without rule, will deduce rules of their own from their previous knowledge of general principles. It appears to me that really correct practice can be found only with men who have enough scope of mind thus in some degree to generalize. Now here, to my mind, lies the obstacle, and as far as I see the insuperable obstacle, to any extensive therapeutical reform. Medicine is essentially a science of observation, and each case demands all the powers of observation that the individual practitioner can bring to it. You cannot reduce the practice of it to a mathematical formula—in a word, you can never make medicine one of the exact sciences. Neither is it to be desired that it should, since its very standing as a science depends upon its complex and uncertain nature. Divest it of this, and reduce the practice of it to the mathematical precision and certainty which the homœopathic humbug pretends to, and it at once becomes degraded to a mere art, a trade, an occupation unworthy to be the object of the higher powers of the mind. I, therefore, not only seem to see the impossibility of a reform in practice to the extent which you appear to contemplate, but I cannot desire it. What then? Are we past amendment? Have we arrived at that state of therapeutical knowledge at which we ought to rest content? Very far from it. I should like to have all our present knowledge accurately retested by competent persons; but I greatly doubt if this is possible. Could this be done, a much greater degree of certainty would attach to our practice. But the really and, to my mind, only feasible plan of reform and improvement will consist in elevating the standard of our professional character, morally and intellectually, and teaching men the value of general principles as a guide through what must, in every individual case, be more or less an experimental procedure."

---

*(V)* "——— June, 1846.

"....In common, I believe, with every one who has at all studied and tested practically the conservative and reparative powers of Nature, the effects of medicines, and the often incalculably vague and doubtful grounds on which the presumed virtues of many or most drugs rest,—the announcements in your article did not entirely take me by surprise.....You have, I conceive, achieved a professional, scientific, and philanthropic duty, of which you may be justly proud; and you must not consider that I say so by way of compliment, but simply to mark my sense of the importance of your labours. In accomplishing any great object or end, such as that which you proposed to yourself, there are two modes, the less creditable of which, alas, is the one too usually adopted. This consists in a compromise, or attempted compromise, of truth with error, of reform with abuse, an introduction of some useful emendations, a retention of many existing imperfections, a fear to take one's stand on broad and ultimate ground. The other mode—that alone worthy of great minds and honest natures, the mode by which the animosities, confusions, sufferings, which unavoidably accompany reforms, are made the least, because the soonest over,—is to occupy at once and from the first, ground coextensive with truth and fact, and nothing short of this. This you have well done in your article. You certainly have not overstepped the due limit. That the projected reformation is directly calculated to affect the interests of mere apothecaries, and also of all quacks in and out of the profession—nay, that it is calculated to curtail the numbers and limit the services of qualified medical men,—cannot be concealed or denied. But whoever would cavil at your article, at your statements, and at the reform you suggest and advocate, on the ground that the adoption of the proposed plans would affect the pecuniary interests, present or future, of himself, his profession, his trade, is, to say the very least, a man whose opinion or opposition may be easily disregarded.

"Will you allow me to state to you my opinion of Dr. Henderson's printed answer to your article? I believe I express the general impression of the profession, when I characterize it as most unsatisfactory and sophistical. I should, moreover, think that homœopathists must be equally dissatisfied with it. Dr. Henderson's homœopathy is certainly not Hahnemann's. His lower dilutions are very little weaker than those now employed by many of *our* way of thinking and prescribing. He adds nothing whatever to the evidence already adduced,—or, perhaps, I should rather say, to the evidence still wanted—to establish the homœopathic principle. The self-complacency—I shall not say, the dogmatism—of his style, in reasoning against a system he so recently held, is somewhat amusing, and confirms the long-made remark that there is no zealot so unmitigated as the proselyte or apostate."

---

*(W)* "———, January 12, 1846.

"The opinions you have offered respecting both homœopathy and allopathy I have long entertained. With regard to homœopathy, I have, publicly and privately, constantly advocated a similar mode of explaining its apparently successful results. My confidence in the allopathic cures, by drugs, was greatly shaken by Louis's 'Recherches.' . . . . . . In speaking or writing respecting the curative or therapeutical powers of drugs, I have a thousand times felt the want of a correct knowledge of the natural history of diseases. How readily do we allopathists detect the want of this knowledge in the homœopathists, yet have overlooked our own ignorance on this point. How is this knowledge to be attained?"

---

*(X)* "——— 8th January, 1846.

"It is long since I waged war under the banner of 'Young Physic,' and I shall most gladly join your ranks and follow your generalship. I have long argued the point with ——, and begged him to try the milk-globules unimpregnated, but without success. I think, in the general opinion of the profession, you will be thought to have gone too far, especially in drug-giving and bill-making England. But in the opinion of the best men, I am sure you will be approved a just judge."

